# Dynamic cerebral autoregulation after confinement in an isolated environment for 14 days

**DOI:** 10.1186/s12199-018-0751-y

**Published:** 2018-12-06

**Authors:** Tomokazu Kato, Ryo Yanagida, Chiharu Takko, Takuya Kurazumi, Natsuhiko Inoue, Go Suzuki, Yojiro Ogawa, Satoshi Furukawa, Ken-ichi Iwasaki

**Affiliations:** 1 Division of Hygiene, Department of Social Medicine, Nihon University School of Medicine, Oyaguchi-Kamicho, Itabashi-ku, Tokyo, 173-8610 Japan; 2 Japan Aerospace Exploration Agency, Ibaraki, 305-8505 Japan

**Keywords:** Cerebral circulation, Space medicine, Transcranial Doppler, Transfer function analysis, Isolated and confined environment, Isolation

## Abstract

**Background:**

To develop human space exploration, it is necessary to study the effects of an isolated and confined environment, as well as a microgravity environment, on cerebral circulation. However, no studies on cerebral circulation in an isolated and confined environment have been reported. Therefore, we investigated the effects of a 14-day period of confinement in an isolated environment on dynamic cerebral autoregulation.

**Methods:**

We participated in an isolation and confinement experiment conducted by the Japan Aerospace Exploration Agency in 2016. Eight healthy males were isolated and confined in a facility for 14 days. Data were collected on the days immediately before and after confinement. Arterial blood pressure waveforms were obtained using a finger blood pressure monitor, and cerebral blood flow velocity waveforms in the middle cerebral artery were obtained using transcranial Doppler ultrasonography for 6 min during quiet rest in a supine position. Dynamic cerebral autoregulation was evaluated by transfer function analysis between spontaneous variability of beat-to-beat mean arterial blood pressure and mean cerebral blood flow velocity.

**Results:**

Transfer function gain in the low- and high-frequency ranges increased significantly (0.54 ± 0.07 to 0.69 ± 0.09 cm/s/mmHg and 0.80 ± 0.05 to 0.92 ± 0.09 cm/s/mmHg, respectively) after the confinement.

**Conclusion:**

The increases observed in transfer function gain may be interpreted as indicating less suppressive capability against transmission from arterial blood pressure oscillation to cerebral blood flow velocity fluctuation. These results suggest that confinement in an isolated environment for 14 days may impair dynamic cerebral autoregulation.

**Trial registration:**

UMIN000020703, Registered 2016/01/22.

## Background

For human space exploration, orthostatic intolerance after spaceflight [1, 2] and visual impairment and intracranial pressure (VIIP) syndrome [3] related to long-term space missions have been critical problems in health care for astronauts. These problems may be associated with alterations in cerebral circulation induced by factors of the space environment such as microgravity and confinement in an isolated environment. A number of simulated microgravity studies regarding the effects of cephalad fluid shift on cerebral circulation have been reported [4, 5]. On the other hand, a number of ground-based studies have shown that confinement in an isolated environment can induce various psychological and physiological changes [6]. However, to our knowledge, no studies have investigated the effects of confinement in an isolated environment on cerebral circulation.

Therefore, in the present study, we investigated the effects of confinement in an isolated environment for 14 days on dynamic cerebral autoregulation using transfer function analysis.

## Methods

### Participants

We participated in an isolation and confinement experiment conducted by the Japan Aerospace Exploration Agency (JAXA). The protocol was approved by the Institutional Ethical Committee of Nihon University School of Medicine (27-7-0-1) and JAXA (79-4-1). We investigated eight healthy males (mean age ± standard deviation 34.3 ± 10.3 years; height 172.9 ± 5.1 cm; weight 70.6 ± 10.3 kg). Written informed consent and medical histories were obtained from all participants before the study began. All participants were screened in regard to their medical history and given physical examinations, including blood pressure measurement. Additional screening included an electrocardiogram (ECG), a psychological test, and an interview. None of the participants had any known medical problems or a history of recreational drug use.

### Equipment

The participants were isolated and confined for 14 days at the “Confinement Environment Adaptation Training Facility” in Tsukuba Space Center. This facility, the details of which have been described by Inoue et al. [7], consists of a habitation module and an experiment module simulating the Japanese experiment module “KIBO” of the International Space Station. This facility has been to evaluate applicants’ behavioral reactions to a stressful environment and to conduct experiments that simulate spaceflight missions for the selection of astronauts [7].

### Procedure

The following restrictions were placed on the participants in the facility. Participants were requested to refrain from leaving the facility or engaging in recreational activities such as drinking alcohol and smoking; communication was limited to telecommunication with researchers in the control room; and meals basically consisted of preserved foods that mimicked astronaut foods. In addition to predetermined daily schedules, several group and personal tasks were assigned, including previously reported tasks simulating the selection of astronaut candidates [7]. No exercise equipment such as treadmills or stationary bikes was provided. Moreover, the schedule of the present experiment did not include exercise sessions. Each participant took part in a daily medical interview through a video conferencing system.

On the days immediately before and after the 14-day confinement period, data for estimating cerebral circulation were measured while participants were lying in a supine position on a comfortable bed. All participants refrained from engaging in heavy exercise and consuming caffeinated or alcoholic beverages for at least 24 h before the measurements. All measurements were conducted in a quiet, environmentally controlled laboratory at an ambient room temperature of 23–26 °C. Heart rate (HR) was measured using a three-lead ECG, and arterial oxygen saturation (SpO_2_) was measured using pulse oximetry (LifescopePT BSM-1763; Nihon Kohden, Tokyo, Japan). Continuous arterial blood pressure waveforms were obtained noninvasively in the middle finger using photoplethysmography (Finometer MIDI; Finapres Medical Systems, Amsterdam, The Netherlands) at the heart level on a beat-to-beat basis. The absolute value of arterial blood pressure was measured at heart level via intermittent arterial blood pressure measured using the oscillometric method with a cuff sphygmomanometer placed over the right brachial artery (Lifescope PT BSM-1763; Nihon Kohden, Tokyo, Japan). Cerebral blood flow (CBF) velocity waveforms in the middle cerebral artery were obtained continuously by transcranial Doppler (TCD) ultrasonography (EZ-Dop; Compumedics Germany GmbH, Sipplingen, Germany) at a depth of 50–60 mm using a 2 MHz probe. This probe was positioned on the right temporal window and fixed at a constant angle, where the highest CBF velocity and appropriate Doppler signal were obtained. To achieve TCD monitoring with high repeatability, we developed a custom probe holder made of a polymer mold to fit the facial bone structure and ear of each participant [8] before taking the first measurements. All TCD measurements were performed by a single experienced expert. After resting in the supine position for at least 15 min, continuous ECG, arterial blood pressure, and CBF velocity waveforms were recorded for 6 min at a sampling rate of 1 kHz using commercial software (Notocord-hem 3.3; Notocord, Paris, France). End-tidal carbon dioxide (ETCO_2_) and respiratory rate were monitored continuously using capnography by nasal cannula (OLG-2800; Nihon Kohden, Tokyo, Japan).

### Data analysis

Steady-state mean arterial blood pressure (MAP) was estimated using the values of intermittent blood pressure measured using the cuff sphygmomanometer. Mean values for steady-state HR and mean CBF velocity (MCBFV) were obtained by averaging the 6-min data segments. SpO_2_, ETCO_2_, and respiratory rate were recorded manually every minute and calculated by averaging the data.

**Fig. 1:**
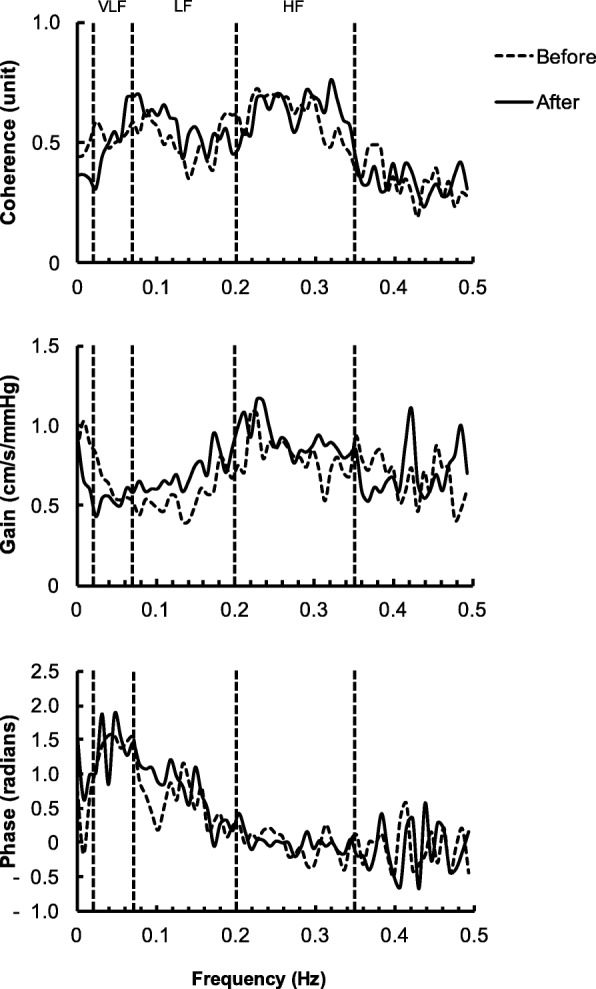
Group-averaged frequency-domain analysis before and after the 14-day confinement period in an isolated environment. Coherence, coherence function; gain, transfer function gain between beat-to-beat MAP and MCBFV; phase, phase between beat-to-beat MAP, and MCBFV; VLF, very low-frequency range (0.02–0.07 Hz); LF, low-frequency range (0.07–0.20 Hz); HF, high-frequency range (0.20–0.35 Hz)

6 min of continuous arterial blood pressure and CBF velocity waveforms were used for spectral and transfer function analyses during spontaneous respiration of room air. The beat-to-beat values of MAP and MCBFV were obtained by integrating analog signals with each cardiac cycle using the PC-based Notocord software. The beat-to-beat values of MAP and MCBFV were then linearly interpolated and resampled at 2 Hz. The MAP and MCBFV time series were first detrended with third-order polynomial fitting using a previously validated algorithm [9, 10]. Therefore, the present analysis applied a 2 Hz resample rate and third-order polynomial fitting to reduce any effects of a gradual slow trend on the 6 min of data. Fast Fourier Transform and transfer function analysis were performed using a Hanning-windowed data segment and then subdivided into 256-point segments with 50% overlap for spectral estimation. This process resulted in five data segments over 6-min data recordings to assess the dynamic pressure-flow relationship. The data analysis was performed using DADiSP software (DSP Development, Cambridge, MA, USA). The spectral power of beat-to-beat MAP and MCBFV variabilities, mean value of coherence, transfer function gain and phase were estimated in the very low- (0.02–0.07 Hz), low- (0.07–0.20 Hz), and high-frequency (0.20–0.35 Hz) ranges (Fig. 1). These frequency ranges were specifically selected to reflect different patterns of the dynamic pressure-flow relationship, as previously identified by transfer function analysis [11–13]. Coherence between 0 and 1 reflects the linear relationship between arterial blood pressure oscillation and CBF velocity fluctuation, with higher coherence indicating a greater linear relationship between the two variables. When coherence is greater than 0.5, transfer function gain and phase are generally considered interpretable indexes. Transfer function gain reflects the capability of the distal cerebral arterioles to suppress transmission from arterial blood pressure oscillation to CBF velocity fluctuation. A larger gain implies that any given change in arterial blood pressure oscillation leads to a larger change in CBF velocity fluctuation, indicating impaired dynamic cerebral autoregulation. Phase reflects the temporal relationship between arterial blood pressure oscillation and CBF velocity fluctuation.

### Statistical analysis

Data are presented as mean ± standard error of the mean. Variables between before and after the 14-day period of confinement were compared using a two-tailed and paired *t* test. A *P* value < 0.05 was considered statistically significant. Statistical analysis was performed using SygmaStat software (version 3.11; Systat Software, San Jose, CA, USA).

## Results

All participants completed the protocol for the 14-day confinement period.

Changes in steady-state hemodynamics and respiratory condition are shown in Table 1. Steady-state HR, MAP, and MCBFV did not change after the 14-day confinement period. In addition, no changes were observed in SpO_2_, ETCO_2_, or respiratory rate.

**Table 1:** Steady-state hemodynamic and respiratory condition

	Before	After	*P* value
HR (beats/min)	62 ± 3	62 ± 3	0.86
MAP (mmHg)	86 ± 2	86 ± 3	0.90
MCBFV (cm/s)	48 ± 4	49 ± 5	0.81
SpO_2_ (%)	97 ± 1	98 ± 0	0.20
ETCO_2_ (mmHg)	40 ± 2	39 ± 1	0.63
RR (breath/min)	15 ± 1	16 ± 1	0.12

Values are means ± SEM. Before, before the 14-day confinement period in an isolated environment; *After* after the 14-day confinement period in an isolated environment, *HR* heart rate, *MAP* mean arterial blood pressure, *MCBFV* mean cerebral blood flow velocity, *SpO*_*2*_ arterial oxygen saturation, *ETCO*_*2*_ end-tidal carbon dioxide, *RR* respiratory rate

**Fig. 2:**
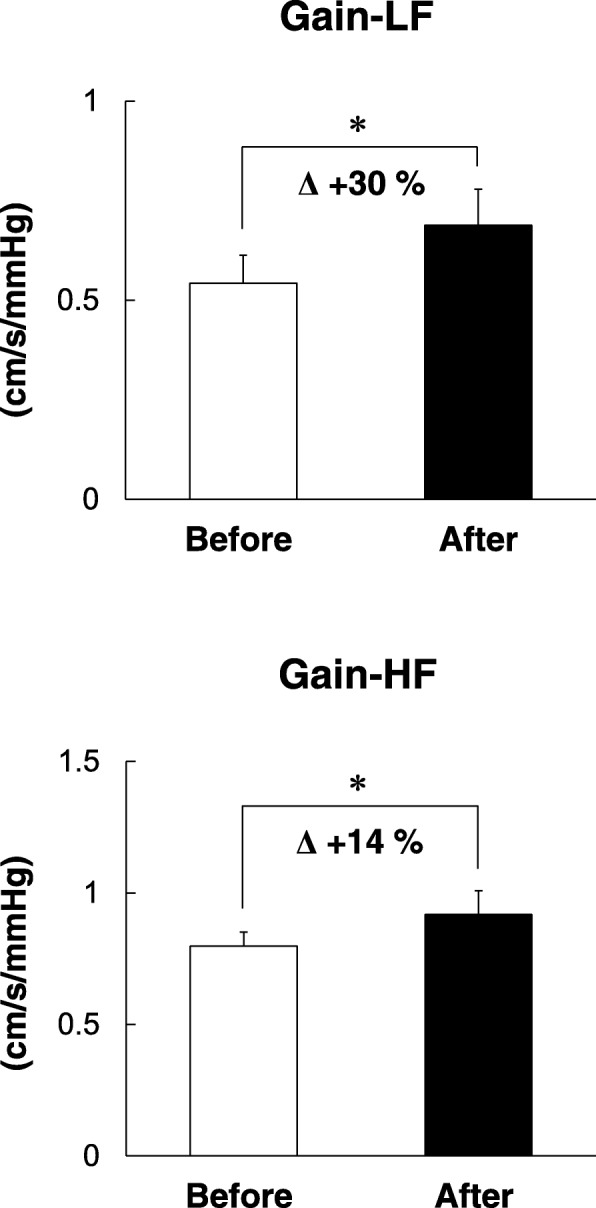
Group-averaged transfer function gain before and after the 14-day confinement period in an isolated environment. *P < 0.05 comparison between before and after the 14-day confinement period in an isolated environment. Δ, percentage change between before and after the 14-day confinement period in an isolated environment. Average percentage changes of transfer function gain were calculated from the percentage changes of each subject

Changes in frequency analysis indexes are shown in Table 2. The group-averaged transfer function analyses between beat-to-beat changes in MAP and MCBFV before and after the 14-day confinement period are shown in Fig. 1. The power of beat-to-beat MAP and MCBFV variability did not change in any frequency ranges. Transfer function gain in the low- and high-frequency ranges increased significantly after the 14-day confinement period (low-frequency *t* (7) = − 2.4, *P* = 0.048; high-frequency *t* (7) = − 2.4, *P* = 0.046). The percentage changes of transfer function gain were 30% in the low-frequency range and 14% in the high-frequency range (Fig. 2). Coherence in these frequency ranges was above 0.5. Phase did not change in these frequency ranges. No indexes of transfer function analysis changed in the very low-frequency range.

**Table 2:** Spectral and transfer function analysis

	Before	After	*P* value
VLF_MAP_ (mmHg^2^)	6.41 ± 1.20	6.61 ± 1.23	0.91
VLF_vel_ (cm^2^/s^2^)	4.30 ± 1.06	3.92 ± 0.96	0.58
CohVLF (unit)	0.52 ± 0.04	0.50 ± 0.04	0.56
GainVLF (cm/s/mmHg)	0.63 ± 0.10	0.53 ± 0.08	0.37
PhaseVLF (radians)	1.41 ± 0.10	1.41 ± 0.17	0.97
LF_MAP_ (mmHg^2^)	2.50 ± 0.45	2.55 ± 0.49	0.93
LF_vel_ (cm^2^/s^2^)	1.19 ± 0.22	1.43 ± 0.20	0.20
CohLF (unit)	0.51 ± 0.06	0.58 ± 0.06	0.18
GainLF (cm/s/mmHg)	0.54 ± 0.07	0.69 ± 0.09*	0.048
PhaseLF (radians)	0.63 ± 0.16	0.81 ± 0.13	0.50
HF_MAP_ (mmHg^2^)	0.25 ± 0.06	0.18 ± 0.04	0.29
HF_vel_ (cm^2^/s^2^)	0.25 ± 0.05	0.23 ± 0.06	0.82
CohHF (unit)	0.61 ± 0.05	0.63 ± 0.06	0.74
GainHF (cm/s/mmHg)	0.80 ± 0.05	0.92 ± 0.09*	0.046
PhaseHF (radians)	− 0.01 ± 0.10	0.01 ± 0.04	0.79

Values are means ± SEM. *Before* before the 14-day confinement period in an isolated environment, *After* after the 14-day confinement period in an isolated environment, *VLF*_*MAP*_ very low-frequency component of mean arterial blood pressure (MAP) variability, *VLF*_*vel*_ very low-frequency component of mean cerebral blood flow velocity (MCBFV) variability, *CohVLF* coherence in the very low-frequency range, *GainVLF* transfer function gain in the very low-frequency range, *PhaseVLF* phase in the very low-frequency range, *LF*_*MAP*_ low-frequency component of MAP variability, *LF*_*vel*_ low-frequency component of MCBFV variability, *CohLF* coherence in the low-frequency range, *GainLF* transfer function gain in the low-frequency range, *PhaseLF* phase in the low-frequency range, *HF*_*MAP*_ high-frequency component of MAP variability, *HF*_*vel*_ high-frequency component of MCBFV variability, *CohHF* coherence in the high-frequency range, *GainHF* transfer function gain in the high-frequency range, *PhaseHF* phase in the high-frequency range. *P<0.05 comparison between before and after the 14-day confinement period in an isolated environment

## Discussion

To the best of our knowledge, no previous studies have reported the effects of an isolated and confined environment on dynamic cerebral autoregulation. Therefore, we studied the effects of a 14-day confinement period in an isolated environment on dynamic cerebral autoregulation using transfer function analysis. The main finding of the present study was that the 14-day confinement period resulted in increased transfer function gain in the low- and high-frequency ranges, despite no changes being observed in steady-state MAP or MCBFV. Increased transfer function gain means that transmission from arterial blood pressure oscillation to CBF velocity fluctuation is amplified with less suppressive capability, suggesting the impairment of dynamic cerebral autoregulation after confinement in an isolated environment; this finding was similar to those in previous studies reporting that increased transfer function gain by hypercapnia impairs cerebral autoregulation [13, 14].

Cerebral autoregulation maintains steady-state CBF by adjusting arteriolar resistance in the brain despite changes in arterial blood pressure. Moreover, the ability of a rapid cerebrovascular response to buffer changes in CBF velocity induced by transient changes in arterial blood pressure is referred to as dynamic cerebral autoregulation. In addition to the assessment of steady-state CBF, transfer function analysis allows the dynamic relationship between arterial blood pressure oscillation and CBF velocity fluctuation to be evaluated. Previous studies have shown that several clinical conditions affect dynamic cerebral autoregulation, but not steady-state MCBFV [15–17]. In the present study, no changes were observed in steady-state MAP or MCBFV obtained by averaging 6 min of data. On the other hand, significant increases were observed in transfer function gain obtained by transfer function analysis after the 14-day confinement period. Thus, similar to previous reports, transfer function analysis detected alterations in dynamic cerebral autoregulation under conditions in which steady-state CBF remained unchanged.

A number of studies have reported that confinement in an isolated environment can lead to various psychological and physiological changes [6]. However, which of the factors constituting the environment induces their changes remains unclear. Although we did not attempt to elucidate the mechanisms underlying altered cerebral autoregulation in the present study, the findings suggest the possibility that cerebral autoregulation is altered by psychological changes owing to confinement in an isolated environment. Further studies are needed to examine this possibility.

In addition, it is possible that the concentration of carbon dioxide inside the facility could have affected dynamic cerebral autoregulation in the preset study. Generally, exposure to high concentrations of carbon dioxide leads to hypercapnia, which induces increased steady-state CBF and impairs dynamic cerebral autoregulation [5]. However, the concentration of carbon dioxide inside the facility in the present study was strictly controlled during the experiment. Therefore, no significant differences in ETCO_2_ or steady-state MCBFV were found between before and after confinement in the present study, suggesting that the concentration of carbon dioxide inside the facility was not likely to influence the impairment of dynamic cerebral autoregulation.

The main limitation of the present study is that the 14-day confinement period took place in a multi-factorial environment. It is assumed that the 14-day confinement period consisted of some factors that might have affected dynamic cerebral autoregulation. As mentioned above, the present study did not attempt to identify the factors underlying the impairment of dynamic cerebral autoregulation. Another limitation is the small number of participants. Conceivably, some of our results might have been different with a larger study population, implying a type I and/or II error.

Another limitation of the present study is that we chose only men as participants for the purpose of simplifying the experimental stress model for confinement in an isolated environment, as some physiological measures, such as cerebral circulation, can be influenced by the menstrual cycle and female sex hormones [18, 19]. The menstrual cycle was considered especially likely to have an effect on cerebral circulation in this experiment because the duration of isolation was 14 days or half of the typical menstrual cycle.

It is well known that some astronauts experience orthostatic intolerance upon return to Earth [1, 2]. The mechanisms responsible for orthostatic intolerance involve multiple factors. Alterations in cerebral autoregulation are considered one of the factors underlying orthostatic intolerance. In fact, cerebral autoregulation was reportedly impaired in astronauts who had experienced orthostatic intolerance after spaceflight [2]. However, which of the factors constituting the space environment impairs cerebral autoregulation has not been completely elucidated. The results of the present study suggest that confinement in an isolated environment may impair dynamic cerebral autoregulation. Therefore, it is possible that an isolated and confined environment is related, at least in part, to orthostatic intolerance upon return to earth. To reveal the mechanisms of orthostatic intolerance, it may be important to examine the effects of confinement in an isolated environment during spaceflight on cerebral circulation. In addition, the present findings can be considered related to medical problems after disasters such as earthquakes, in which victims suddenly find themselves in an isolated and confined environment.

## Conclusion

The results of the present study showed that transfer function gain in the low- and high-frequency ranges increased after the 14-day confinement period. Increased transfer function gain would indicate that transmission from arterial blood pressure oscillation to CBF velocity fluctuation is amplified with less suppressive capability. Therefore, the present results suggest that confinement in an isolated environment may impair dynamic cerebral autoregulation.

## Abbreviations

CBF: Cerebral blood flow
ECG: Electrocardiogram
ETCO_2_: End-tidal carbon dioxide
JAXA: Japan Aerospace Exploration Agency
MAP: Mean arterial blood pressure
MCBFV: Mean cerebral blood flow velocity
SpO_2_: Arterial oxygen saturation
TCD: Transcranial Doppler
VIIP: Visual impairment and intracranial pressure
